# Practice patterns of strong opioid recommendations by palliative care consultation services in German hospitals (online survey)

**DOI:** 10.1186/s12904-026-02266-y

**Published:** 2026-07-31

**Authors:** Evelyn Mueller, Susanne Gahr, Annette Schnell, Christoph Aulmann, Carmen Roch

**Affiliations:** 1 Interdisciplinary Center for Palliative Medicine, Department of Radiation Oncology, CCC Mainfranken, University Hospital Wuerzburg, Josef-Schneider-Str. 11, Würzburg, 97080 Germany; 2 Department of Palliative Medicine, CCC Erlangen-EMN, University Hospital Erlangen, Friedrich-Alexander-University Erlangen-Nürnberg (FAU), Universitaetsstr. 21/23, Erlangen, 91054 Germany; 3 Center for Palliative Medicine, CCC Regensburg, University Hospital Regensburg, Franz-Josef-Strauß-Allee 11, Regensburg, 93053 Germany; 4 Palliative Medicine, Faculty of Medicine, University of Augsburg, Stenglinstr. 2, Augsburg, 86156 Germany; 5 Comprehensive Cancer Center Alliance WERA (CCC WERA), Bavaria, Germany

**Keywords:** Analgesics, Opioid, Palliative care, Palliative medicine, Specialist consultation

## Abstract

**Background:**

Strong opioids are central to effective symptom control in palliative care. In cases of complex symptom burden, primary care teams frequently rely on palliative care consultation services (PCCSs) for expert guidance. This survey explored the recommendations issued by hospital-based PCCS teams regarding the use of strong opioids in patients with advanced disease (e.g. clinical indications, opioid agent).

**Methods:**

A closed online survey was conducted via the SoSci Survey platform. All PCCS teams registered with the German Association for Palliative Medicine were invited to participate in November 2024, with a reminder sent three weeks later.

**Results:**

Thirty-nine PCCS teams participated (21 university hospitals, 18 general hospitals). All respondents agreed on the indication for strong opioids in cancer-related pain and dyspnea. Differences emerged regarding the use of opioids in patients with non-malignant conditions: although most PCCS considered opioids appropriate for dyspnea associated with pulmonary (*n* = 37) or cardiac disease (*n* = 33), non-cancer pain was less consistently endorsed (*n* = 20). Hydromorphone and morphine were the most commonly used opioids (> 30 services reporting frequent use), whereas the use of fentanyl, oxycodone, and buprenorphine varied widely across services.

**Conclusions:**

There is consensus regarding the use of opioids in patients with cancer-related pain and dyspnea. The differing approaches to their use for symptoms caused by non-malignant conditions suggest varying clinical interpretations and underscore the need for further research and clearer guidelines.

**Supplementary Information:**

The online version contains supplementary material available at 10.1186/s12904-026-02266-y.

## Introduction

Effective symptom control is a central principle of palliative care, with pain relief considered a key prerequisite for a “good death” and consistently ranked among the highest priorities of patients with advanced disease [1–3]. Specialist Palliative Care (SPC), including hospital-based palliative care consultation services (PCCS), plays an essential role in the management of complex symptom burden working collaboratively with primary treatment teams to provide individualized support [4]. PCCS teams aim to ensure that patients who are not treated on dedicated palliative care units receive the same standards of symptom management throughout their hospital stay [5].

Pain is one of the most frequent and distressing symptoms among patients with advanced cancer [3]. Despite the availability of effective treatments, evidence shows that there is often a need für better pain management in this population [3, 6]. The integration of PCCSs has been associated with improvements in pain intensity and symptom burden, suggesting that specialized consultation contributes meaningfully to clinical outcomes [7–10]. Current German guidelines on palliative care for patients with incurable cancer are adapted from the European Association for Palliative Care (EAPC) recommendations and are grounded in WHO analgesic standards [11–13].

Meanwhile, the WHO distinguishes mainly between weak and strong opioids without strict reference to the original analgesic ladder. Despite this change, the classification continues to provide valuable framework for clinical practice. Consequently, strong opioids constitute the foundation of effective pain management, particularly for cancer-related pain, and are equally indispensable for the management of distressing symptoms, including dyspnea, in patients with both cancer and non-malignant conditions [7, 9].

Alongside pain, dyspnea represents another highly burdensome symptom in patients with advanced disease, frequently associated with significant functional impairment and psychological distress [8, 14]. Although the underlying causes of chronic dyspnea are often well defined, therapeutic options may be limited once symptoms become refractory and further optimization of cause-related treatment proves ineffective. In clinical palliative practice, opioids are commonly used for refractory breathlessness. The German palliative care guideline for patients with incurable cancer recommends its use for dyspnea [11]. However, there is only limited and disease-specific evidence [8, 14–16]. The use of morphine for this indication has received regulatory approval in only a few countries; in most countries, physicians must resort to off-label use in their daily practice [17, 18]. Decisions regarding substance choice and route of administration must therefore consider both clinical evidence and regulatory constraints [11].

Prescribing opioids is highly regulated due to their potential for severe adverse events, and risk of non-medical use [19, 20]. German legal frameworks distinguish between an opioid prescription issued directly by a PCCS physician versus a clinical recommendation addressed to the primary treating team. Direct prescribing transfers full medical and legal responsibility to the PCCS physician, while recommendations rely on implementation by other clinicians [21]. Whether PCCS physicians recommend or directly prescribe opioids may influence their roles and workflows and could, in turn, affect treatment decisions [22, 23].

The aim of this survey was to systematically explore the recommendations issued by hospital-based PCCS teams regarding the use of strong opioids in patients with advanced disease in Germany. Specifically, the study sought to analyze the recommended opioid agents, clinical indications, and routes of administration, as well as possible differences between university and general hospitals in their recommendations.

## Materials and methods

### Study design

A closed online survey was conducted among palliative care consultation services (PCCS) in German hospitals using the SoSci Survey platform [24]. Reporting follows the CHERRIES checklist [25].

### Sample and survey administration

All PCCS listed in the directory of the German Association for Palliative Medicine in October 2024 or included in the university palliative care working group mailing list were eligible. The survey was to be completed by (senior) medical staff with at least one year of PCCS experience or jointly by team members. Each PCCS could submit one completed questionnaire. A database of PCCS email contacts was compiled; outdated addresses were updated via online searches, and inactive services were removed. Invitations to participate were emailed on 14 November 2024, followed by a reminder on 3 December 2024. The invitation included information on study purpose, procedures, expected time, data handling, and anonymity. Each facility received a personalized link permitting a single submission; links supported pausing and resuming but were not tracked to maintain anonymity. The survey closed on 31 December 2024. Participants were given the option to voluntarily provide an email address to receive the study results; these were stored separately. No incentives were offered.

### Questionnaire development and questionnaire structure

The questionnaire was developed by CR and EM based on literature, clinical experience, and discussions with the CCC WERA Palliative Medicine working group (Comprehensive Cancer Centers Würzburg/Erlangen/Regensburg/Augsburg specifically for this research project. The dataset has previously been used for a separate publication addressing the first study question; the present manuscript reports on a second, independent research question [26]. Pre-testing involved nine palliative care physicians and one specialist nurse from six German and one Swiss site. Pilot testing included: (a) content validation, (b) cognitive interviews (think-aloud and probing) to assess clarity, relevance, and scaling, and (c) testing of technical functionality and usability. The final survey comprised four thematic sections across six online pages: PCCS opioid recommendations (1 page, 4 items), implementation of recommendations (2 pages, 5 items), needs and potential improvement measures (1 page, 3 items), PCCS characteristics (1 page, 7 items), and participant characteristics (1 page, 4 items). Four items included prompts for missing responses; continuation without answering was possible. Responses could be revised at any point.

### Data management and analysis

Data were exported from ScoSci Survey [24] in SPSS format and analyzed using IBM SPSS 29 [27]. Surveys with > 30% missing data on closed questions were excluded. No time limit for completion was applied. Group differences were analyzed using Chi-square or Kruskal-Wallis H tests with post-hoc Dunn tests, depending on variable type. The significance level was set at 0.05 (two-tailed). Given the exploratory approach, no Bonferroni correction was applied. Missing data were reported transparently.

## Results

### Participation and sample

The initial contact database comprised 95 PCCS. Ten email addresses were invalid, resulting in 85 successfully delivered invitations (26 university hospital PCCS, 59 general hospital PCCS). The survey link was accessed by 47 PCCS (view rate 55%). Of these, 41 initiated the questionnaire (participation rate 87%). Two responses were excluded due to > 30% missing data, yielding 39 evaluable datasets (completion rate 95%). Overall, evaluable data were obtained from 39 of 85 contacted PCCS (46%), including 18 of 59 general hospital PCCS (31%) and 21 of 26 university hospital PCCS (81%).

### Characteristics of Palliative Care Consultation Services (PCCS)

The distribution between university hospitals (*n* = 21) and non-university hospitals (*n* = 18) is nearly balanced. Most services are located in large hospitals with more than 500 beds and have been established for more than two years (Table 1). Marked differences are evident between PCCS in university and non-university hospitals: university hospitals significantly more often have a higher bed capacity, are almost consistently affiliated with a Comprehensive Cancer Center, and invariably operate a dedicated inpatient palliative care unit. Some non-university PCCS follow different practice structures: eight of 18 non-university services report issuing opioid prescriptions directly rather than only providing recommendations, and two report acting as external providers offering consultation services to hospitals. In all cases, the survey was completed by board-certified palliative care physicians, mostly in senior positions.

**Table 1:** Characteristics of PCCS and respondents (Frequency n, Percentage %)

	All participants	University Hospital	General Hospital	Significance test*
PCCS is an…				
Palliative care consultation service				
internal structure at a hospital	37 (95)	21 (100)	16 (89)	X^2^(1) = 2.46; *p* = 0.117
external, consultations at hospitals	2 (5)	0 (0)	2 (11)	
Hospital size				
Up to 299 beds	5 (13)	0 (0)	5 (28)	U = 31.50; Z = -5.09; *p* < 0.001
300 to 499 beds	3 (8)	0 (0)	3 (17)	
500 to 699 beds	7 (18)	0 (0)	7 (39)	
700 to over 1000 beds	24 (62)	21 (100)	3 (17)	
Comprehensive Cancer Center				
Yes	22 (56)	19 (90)	3 (17)	X^2^(1) = 20.44; *p* < 0.001
No	16 (41)	2 (10)	14 (78)	
No answer	1 (3)	0 (0)	1 (6)	
Palliative care unit				
Yes	30 (77)	21 (100)	9 (50)	X^2^(1) = 13.65; *p* < 0.001
No	9 (23)	0 (0)	9 (50)	
Recommendation vs. prescription of opioids				
Recommendation	31 (79)	21 (100)	10 (56)	X^2^(1) = 11.74; *p* < 0.001
Prescription	8 (21)	0 (0)	8 (44)	
Respondent				
Gender				
Female	30 (77)	19 (90)	11 (61)	X^2^(1) = 47.08; *p* = 0.03
Male	9 (23)	2 (10)	7 (39)	
Position				
Specialist doctor (leading position)	31 (79)	16 (76)	15 (83)	X^2^(1) = 0.30; *p* = 0.582
Specialist doctor (no leading position)	8 (21)	5 (24)	3 (17)	
Specialist for…				
internal medicine	16 (41)	7 (33)	9 (50)	X^2^(2) = 24.62; *p* = 0.292
anaesthesiology	2 (5)	2 (10)	0 (0)	
general medicine	21 (54)	12 (57)	9 (50)	
Additional qualification (more than one possible)				
Palliative medicine	39 (100)	21 (100)	18 (100)	--
Emergency medicine	12 (31)	5 (24)	7 (39)	X^2^(1) = 10.35; *p* = 0.309
Pain therapy	8 (21)	4 (19)	4 (22)	X^2^(1) = 0.06; *p* = 0.807

* Chi-square test (nominal data) or Kruskal-Wallis H-test (ordinal data); significance level *p* < 0.05 (two-sided)

### Opioid recommendations

Proportions of patients with recommendations on strong opioids vary considerably between services (range 40–100%). One service indicated that 21–40% of patients receive recommendations, 10 services reported a proportion of 41–60%, 19 services 61–80%, and 9 services 81–100% of patients (Additional File 1, Tab S1).

**Fig. 1:**
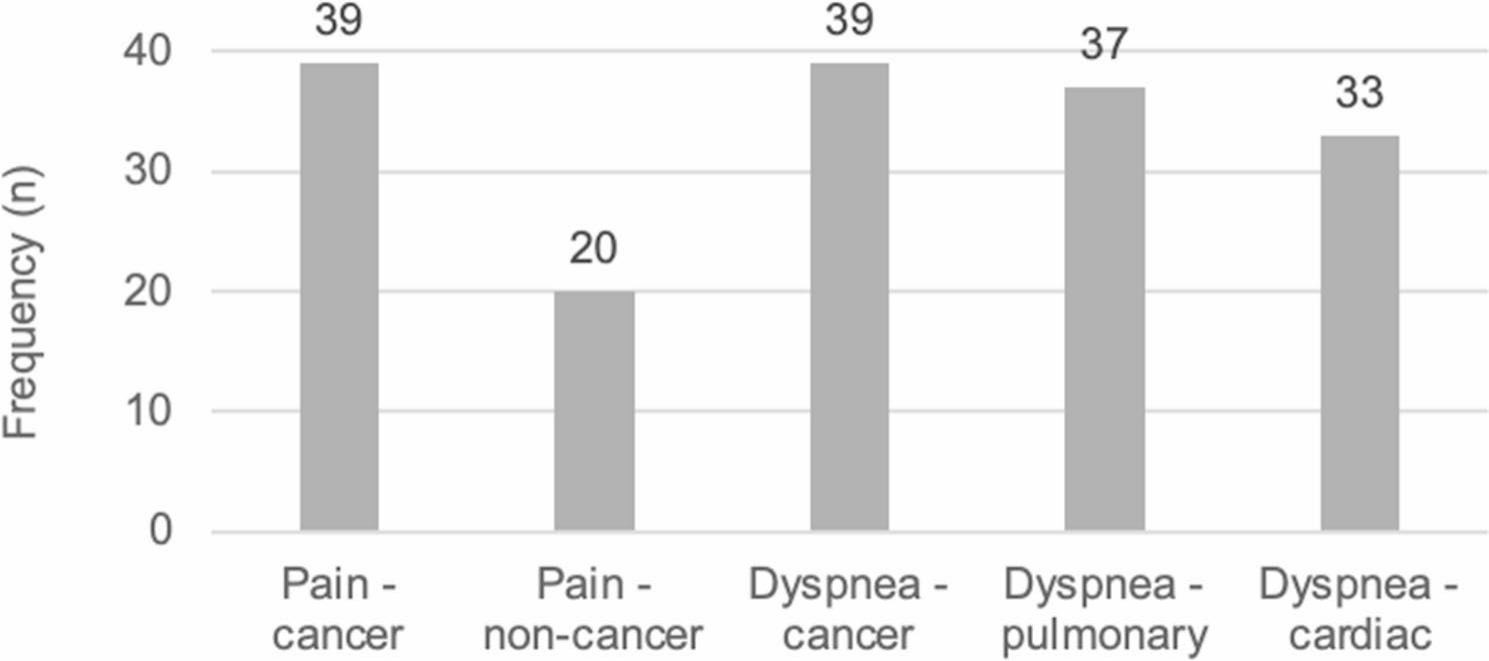
Frequency of agreement on indications for strong opioids by PCCS (see Additional File 1, Tab S2)

All respondents agreed on the indication for strong opioids in cancer-related pain and dyspnea. Differences emerged regarding patients with non-malignant conditions: more than half of respondents considered strong opioids appropriate for non-cancer pain and dyspnea associated with pulmonary or cardiac diseases, the indication for pain in patients with non-malignant diseases was endorsed less frequently (see Fig. 1).

**Fig. 2:**
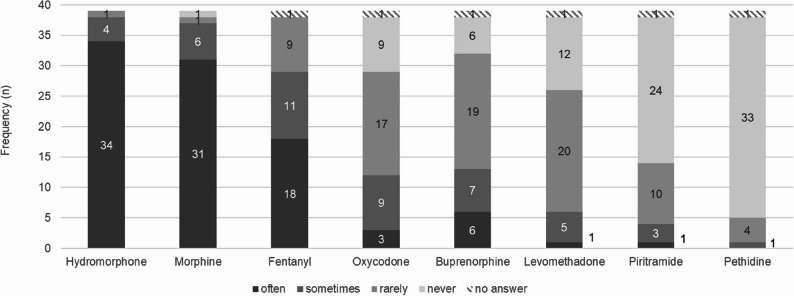
Frequency of use of different strong opioids by PCCS (see Additional File 1, Tab S3)

The two most commonly used opioids are hydromorphone and morphine, although some palliative care services report using them only occasionally (Fig. 1). Fentanyl, oxycodone, and buprenorphine are regularly used by several palliative care services, whereas levomethadone, piritramide, a synthetic opioid analgesic commonly used for postoperative pain-management, and pethidine are assigned only a minor role (Fig. 2).

**Fig. 3:**
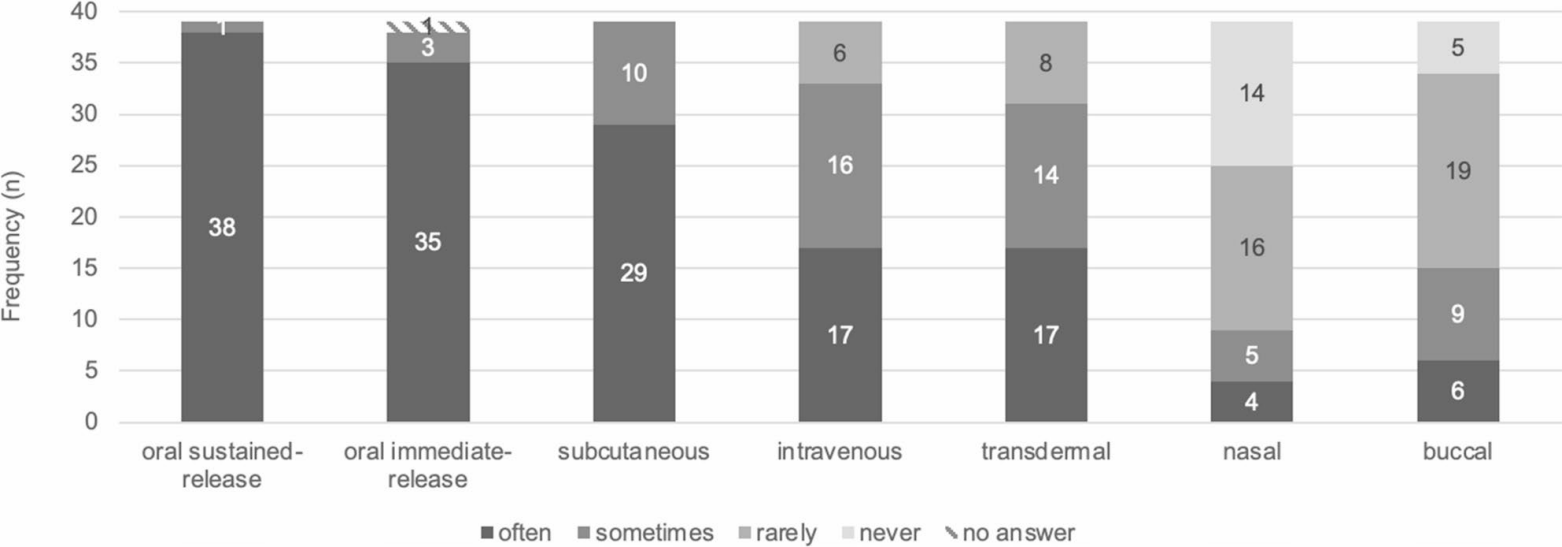
Frequency of different routes of administration of strong opioids (see Additional File 1, Tab S4)

Regarding the routes of administration of strong opioids, orally (both sustained and immediate-release) administered formulations predominate, followed by subcutaneous, intravenous, and transdermal administration. In contrast, nasal and buccal applications play a relevant role in only a few palliative care services (Fig. 3).

University and general hospitals did not differ systematically in recommended opioid agents, clinical indications or routes of administration. The only statistically significant difference concerned the use of oral immediate-release opioids (U = 147.00; Z = -1.98; *p* = 0.048). Whereas all university hospital PCCS reported frequent use, three general hospital PCCS reported using these formulations only sometimes, explaining the observed difference (Additional File 1, Tab S1-S4).

## Discussion

### Recommendations and indications

The near-universal use of strong opioids for cancer-related pain in our sample aligns with WHO recommendations and the German S3 Guideline for Palliative Care. In contrast, only half of the respondents see a medical indication for potent opioids in cases of non-cancer-related pain. The data thus reflect the current controversies: Recent evidence suggests that the WHO analgesic ladder is overrated [7], particularly for chronic non-cancer pain [28]. The S3 Guideline on long-term opioid therapy therefore mandates reassessment after three months, which may explain respondents´ reluctance to use opioids in patients with non-malignant diseases [29]. Nevertheless, the WHO classification system does not differentiate between nociceptive and neuropathic pain and, even with the addition of level 4, offers only limited guidance to inexperienced users [30].

The majority of respondents recommends opioids for refractory dyspnea, despite limited evidence: The indication for opioid use for dyspnea remains particularly controversial. In non-malignant conditions such as heart failure and COPD (chronic obstructive pulmonary disease), recent studies have not demonstrated consistent evidence of benefit other than end-of-life settings [14, 31]. Even in patients with cancer, robust RCTs supporting opioids other than morphine for dyspnea relief are lacking [32]. Furthermore, guidelines addressing non-malignant conditions rarely discuss refractory dyspnea; the S3 Guideline for Palliative Care is a notable exception as it provides symptom-oriented recommendations. This gap is clinically relevant, as dyspnea in non-patients with non-malignant diseases is among the most frequent and burdensome end-of-life symptoms, with a symptom burden comparable to that observed in cancer [33, 34]. The absence of clear, diagnosis-independent recommendations carries a risk of structural undertreatment and highlights the need for evidence-based strategies to manage refractory dyspnea- regardless of underlying disease.

### Use of different opioids

In our survey, the responding PCCS most commonly used opioids were the µ-receptor agonists hydromorphone and morphine. Their predominance is consistent with current evidence and clinical practice, as both agents are available in oral and parenteral forms, allowing uninterrupted therapy when patient can no longer swallow [35]. In addition, fentanyl is frequently used, while buprenorphine, oxycodone, and other active ingredients play a role in only a very small number of PCCS.

Across multiple randomized controlled trials, no consistent or clinically meaningful differences have been demonstrated in the analgesic efficacy of commonly used opioids in palliative care – including morphine, oxycodone, and hydromorphone – that would support a strong preference for any specific agent [7, 12].

For other factors influencing the choice of opioids include pharmacological restrictions, drug availability, costs, individual contraindications, treatment team preferences, and patient’s reluctance [36, 37]. Consequently, such clinical situations often require the use of opioids with less reliance on renal elimination, such as hydromorphone, although even hydromorphone metabolites may accumulate in advanced renal failure.

### Routes of administration

In our cohort; oral formulations were most frequently used, followed by subcutaneous administration. Transdermal opioids were used less frequently, despite being recommended in some clinical guidelines as potential first-line alternatives and as options for opioid rotation.

The classical indications for opioid therapy in palliative care are often characterized by acute symptom onset. Oral formulations are commonly used in patients who are able to swallow and wish to maintain a high degree of autonomy. In clinical practice, opioids are frequently administered subcutaneously or intravenously to enable rapid dose titration and prompt symptom relief [38]. Because of their delayed onset of action—for example, transdermal fentanyl requires approximately 12–24 h to achieve therapeutic plasma concentrations [39]—transdermal opioid formulations are primarily used in patients with stable and well-controlled symptoms.

The route of administration may influence the potential for non-medical opioid use [40]. However, these concerns are mostly negligible in palliative care settings, particularly when patients can no longer take oral medications, when rapid symptom control is required, or when a limited life expectancy significantly reduces both the risk and clinical relevance of misuse [41].

### Characteristics of the palliative care services

Most of the palliative care services we surveyed recommend opioids but do not prescribe them. PCCS in smaller general hospitals - including those that prescribe opioids directly - report no significant differences in opioid use compared with university hospitals. Opioid prescribing requires not only careful clinical assessment but also short and long-term treatment planning, including repeated evaluation of efficiency and adverse effects [42]. Within the context of palliative care consultation, it is therefore essential to clarify in advance who is responsible for monitoring and managing typical opioid related adverse effects such as sedation, respiratory depression, nausea, vomiting, and urinary retention to ensure safe and coordinated care [35].

### Strengths and limitations

All participants were experienced, well-trained PCCS physicians and thereby likely to be able to evaluate the topic of the survey. Participation and completion rates were high among university hospital PCCS, however, significantly lower for general hospital PCCS. This is likely to be due to email invitations being classified as spam, which we know happened in some cases. However, transferability to other countries or settings may be limited due to differences in regulations, framework conditions and availability of strong opioids.

### Conclusion

The results of the survey on opioid recommendations by palliative care services largely reflect the evidence base: clear evidence corresponds to broad agreement on opioid recommendations, while unclear evidence corresponds to significant variability. However, it also becomes clear that the specific requirements of palliative care situations are significant. For example, even without sufficient evidence, opioids are prescribed for refractory dyspnea caused by e.g. cardiac or neurological conditions, since no other treatment options exist, long-term side effects become less significant, and acute suffering is often alleviated.

This highlights the need for robust research on the efficacy, safety and optimal dosing of strong opioids in patients with advanced, incurable disease – especially for dyspnea and pain caused by non-malignant conditions. Clear, evidence-based guidelines are required to support clinical decision-making, reduce practice variability, and ensure effective symptom control.

## Supplementary Information

Supplementary Material 1: Detailed statistics.

## Data Availability

The datasets analyzed during the current study are available from the corresponding author on reasonable request.

## Abbreviations

CCC: Comprehensive Cancer Center
CCC WERA: Alliance Comprehensive Cancer Center Würzburg, Erlangen, Regensburg, Augsburg
EAPC: European Association for Palliative Care
M3G: Morphine 3 Glucuronide
M6G: Morphine 6 Glucuronide
PCCS: Palliative Care Consultation Service
SPC: Specialist Palliative Care
WHO: World Health Organization
