# Supplementary material for: Practice patterns of strong opioid recommendations by palliative care consultation services in German hospitals (online survey)
Source: BMC Palliat Care. 2026 Jul 31;25:223. doi: 10.1186/s12904-026-02266-y (PMC13428421; doi:10.1186/s12904-026-02266-y)
Supplement: Supplementary file 1 — Supplementary Material 1: Detailed statistics. [file 12904_2026_2266_MOESM1_ESM.docx]

# **Additional File – Detailed Statistics**

Tab. S1: Proportion of patients with strong opioid recommendations (n (%))

|  | **All participants** | **University Hospital** | **General Hospital** | **Significance test** |
| --- | --- | --- | --- | --- |
| 0 - 20 % | 0 (0) | 0 (0) | 0 (0) | U = 179.00; Z = -0.31; p = 0.761 |
| 21 - 40 % | 1 (3) | 0 (0) | 1 (6) |  |
| 41 - 60 % | 10 (26) | 6 (29) | 4 (22) |  |
| 61 - 80 % | 19 (49) | 11 (52) | 8 (44) |  |
| 81 - 100 % | 9 (23) | 4 (19) | 5 (28) |  |

Tab. S2: Agreement with indications for strong opioids (n answering ‚yes‘(%))

|  | **All participants** | **University Hospital** | **General Hospital** | **Significance test*** |
| --- | --- | --- | --- | --- |
| Pain – non cancer | 20 (51) | 10 (48) | 10 (56) | X^2^(1) = 0.24; p = 0.621 |
| Pain - cancer | 39 (100) | 21 (100) | 18 (100) | -- |
| Dyspnea – cancer | 39 (100) | 21 (100) | 18 (100) | -- |
| Dyspnea - pulmonary | 37 (95) | 20 (95) | 17 (94) | X^2^(1) = 0.01; p = 0.911 |
| Dyspnea - cardiac | 33 (85) | 18 (86) | 15 (83) | X^2^(1) = 0.04; p = 0.837 |

Tab. S3: Choice of opioid (n (%))

|  | **All participants** | **Univ. Hospital** | **Gen. Hospital** | **Significance test*** |
| --- | --- | --- | --- | --- |
| Morphine | | | | |
| Never | 1 (3) | 1 (5) | 0 (0) | U = 146.50; Z = -1.70; p = 0.089 |
| Rarely | 1 (3) | 0 (0) | 1 (6) |  |
| Sometimes | 6 (15) | 1 (5) | 5 (28) |  |
| Often | 31 (79) | 19 (90) | 12 (67) |  |
| Hydromorphone | | | | |
| Never | 0 (0) | 0 (0) | 0 (0) | U = 177.00; Z = -0.58; p = 0.56 |
| Rarely | 1 (3) | 1 (5) | 0 (0) |  |
| Sometimes | 4 (10) | 1 (5) | 3 (17) |  |
| Often | 34 (87) | 19 (90) | 15 (83) |  |
| Fentanyl | | | | |
| Never | 0 (0) | 0 (0) | 0 (0) | U = 169.00; Z = -0.30; p = 0.763 |
| Rarely | 9 (23) | 6 (29) | 3 (17) |  |
| Sometimes | 11 (28) | 5 (24) | 6 (33) |  |
| Often | 18 (46) | 10 (48) | 8 (44) |  |
| No answer | 1 (3) | 0 (0) | 1 (6) |  |
| Oxycodone | | | | |
| Never | 9 (23) | 6 (29) | 3 (17) | U = 159.00; Z = -0.61; p = 0.543 |
| Rarely | 17 (44) | 9 (43) | 8 (44) |  |
| Sometimes | 9 (23) | 4 (19) | 5 (28) |  |
| Often | 3 (8) | 2 (10) | 1 (6) |  |
| No answer | 1 (3) | 0 (0) | 1 (6) |  |
| Buprenorphin | | | | |
| Never | 6 (15) | 2 (10) | 4 (22) | U = 127.50; Z = -1.61; p = 0.107 |
| Rarely | 19 (49) | 10 (48) | 9 (50) |  |
| Sometimes | 7 (18) | 4 (19) | 3 (17) |  |
| Often | 6 (15) | 5 (24) | 1 (6) |  |
| No answer | 1 (3) | 0 (0) | 1 (6) |  |
| Levomethadone | | | | |
| Never | 12 (31) | 7 (33) | 5 (28) | U = 165.00; Z = -0.44; p = 0.662 |
| Rarely | 20 (51) | 9 (43) | 11 (61) |  |
| Sometimes | 5 (13) | 5 (24) | 0 (0) |  |
| Often | 1 (3) | 0 (0) | 1 (6) |  |
| No answer | 1 (3) | 0 (0) | 1 (6) |  |
| Piritramide | | | | |
| Never | 24 (62) | 14 (67) | 10 (56) | U = 172.00; Z = -0.22; p = 0.823 |
| Rarely | 10 (26) | 4 (19) | 6 (33) |  |
| Sometimes | 3 (8) | 2 (10) | 1 (6) |  |
| Often | 1 (3) | 1 (5) | 0 (0) |  |
| No answer | 1 (3) | 0 (0) | 0 (0) |  |
| Pethidine | | | | |
| Never | 33 (85) | 19 (90) | 14 (78) | U = 163.00; Z = -0.78; p = 0.438 |
| Rarely | 4 (10) | 2 (10) | 2 (11) |  |
| Sometimes | 1 (3) | 0 (0) | 1 (6) |  |
| No answer | 1 (3) | 0 (0) | 1 (6) |  |

Tab. S4: Routes of Administration of WHO Step III Opioids (n (%))

|  | **All participants** | **University Hospital** | **General Hospital** | **Significance test*** |
| --- | --- | --- | --- | --- |
| Oral sustained-release | | | | |
| Never | 0 (0) | 0 (0) | 0 (0) | U = 178.50; Z = -1.08; p = 0.28 |
| Rarely | 0 (0) | 0 (0) | 0 (0) |  |
| Sometimes | 1 (3) | 0 (0) | 1 (6) |  |
| Often | 38 (97) | 21 (100) | 17 (94) |  |
| Oral immediate-release | | | | |
| Never | 0 (0) | 0 (0) | 0 (0) | U = 147.00; Z = -1.98; p = 0.048 |
| Sometimes | 3 (8) | 0 (0) | 3 (17) |  |
| Often | 35 (90) | 21 (100) | 14 (78) |  |
| No answer | 1 (3) | 0 (0) | 1 (6) |  |
| Subcutaneous | | | | |
| Never | 0 (0) | 0 (0) | 0 (0) | U = 157.50; Z = -1.17; p = 0.241 |
| Rarely | 0 (0) | 0 (0) | 0 (0) |  |
| Sometimes | 10 (26) | 7 (33) | 3 (17) |  |
| Often | 29 (74) | 14 (67) | 15 (83) |  |
| Intravenous | | | | |
| Never | 0 (0) | 0 (0) | 0 (0) | U = 128.50; Z = -1.85; p = 0.064 |
| Rarely | 6 (15) | 2 (10) | 4 (22) |  |
| Sometimes | 16 (41) | 7 (33) | 9 (50) |  |
| Often | 17 (44) | 12 (57) | 5 (28) |  |
| Transdermal | | | | |
| Never | 0 (0) | 0 (0) | 0 (0) | U = 161.50; Z = -0.83; p = 0.404 |
| Rarely | 8 (21) | 3 (14) | 5 (28) |  |
| Sometimes | 14 (36) | 8 (38) | 6 (33) |  |
| Often | 17 (44) | 10 (48) | 7 (39) |  |
| Nasal | | | | |
| Never | 14 (36) | 8 (38) | 6 (33) | U = 172.50; Z = -0.50; p = 0.621 |
| Rarely | 16 (41) | 9 (43) | 7 (39) |  |
| Sometimes | 5 (13) | 2 (10) | 3 (17) |  |
| Often | 4 (10) | 2 (10) | 2 (11) |  |
| Buccal | | | | |
| Never | 5 (13) | 2 (10) | 3 (17) | U = 169.50; Z = -0.59; p = 0.555 |
| Rarely | 19 (49) | 10 (48) | 9 (50) |  |
| Sometimes | 9 (23) | 6 (29) | 3 (17) |  |
| Often | 6 (15) | 3 (14) | 3 (17) |  |
